# Thermally Conductive and Electrically Insulating Polymer-Based Composites Heat Sinks Fabricated by Fusion Deposition Modeling

**DOI:** 10.3390/polym16030432

**Published:** 2024-02-04

**Authors:** Simone Bagatella, Annacarla Cereti, Francesco Manarini, Marco Cavallaro, Raffaella Suriano, Marinella Levi

**Affiliations:** 1 Department of Chemistry, Materials and Chemical Engineering “Giulio Natta”, Politecnico di Milano, Piazza Leonardo Da Vinci 32, 20133 Milan, MI, Italy; marco.cavallaro@polimi.it (M.C.); raffaella.suriano@polimi.it (R.S.); marinella.levi@polimi.it (M.L.); 2 LATI S.p.A., Via Baracca 7, 21040 Vedano Olona, VA, Italy; acereti@it.lati.com (A.C.); fmanarini@it.lati.com (F.M.)

**Keywords:** polymer-matrix composites, boron nitride, thermal conductivity, heat sinks, thermal management, fusion deposition modeling (FDM)

## Abstract

This study explores the potential of novel boron nitride (BN) microplatelet composites with combined thermal conduction and electrical insulation properties. These composites are manufactured through Fusion Deposition Modeling (FDM), and their application for thermal management in electronic devices is demonstrated. The primary focus of this work is, therefore, the investigation of the thermoplastic composite properties to show the 3D printing of lightweight polymeric heat sinks with remarkable thermal performance. By comparing various microfillers, including BN and MgO particles, their effects on material properties and alignment within the polymer matrix during filament fabrication and FDM processing are analyzed. The characterization includes the evaluation of morphology, thermal conductivity, and mechanical and electrical properties. Particularly, a composite with 32 wt% of BN microplatelets shows an in-plane thermal conductivity of 1.97 W m^−1^ K^−1^, offering electrical insulation and excellent printability. To assess practical applications, lightweight pin fin heat sinks using these composites are designed and 3D printed. Their thermal performance is evaluated via thermography under different heating conditions. The findings are very promising for an efficient and cost-effective fabrication of thermal devices, which can be obtained through extrusion-based Additive Manufacturing (AM), such as FDM, and exploited as enhanced thermal management solutions in electronic devices.

## 1. Introduction

With the development of 5G systems, electronic devices are becoming more powerful and highly compact. This places a significant demand on heat removal efficiency [1,2,3]. If the thermal conductivity of the casing material is not high enough, the heat generated by the electronic devices cannot be effectively dissipated, and the system’s stability could be affected [4,5,6]. Indeed, it is well known that the failure rate of electronic devices increases exponentially with the increase in electronic devices’ temperature [7]. Therefore, to address this challenge, there is a growing demand for composite materials with improved thermal conductivity and electrical insulation ability. These materials can effectively remove heat from electronic devices and maintain them at a safe operating temperature, while electrical insulation prevents short circuits and other electrical hazards [8].

Improving thermal management entails enhancing the heat transfer rate through two primary means: either by expanding the surface area to optimize device topology or by improving the thermal conductivity of a heat sink attached to an electronic device, thereby enhancing the material properties. Among the most common equipment for thermal management, heat sinks and heat exchangers are some of the most common solutions. Conventional heat sinks and heat exchangers are typically made of metallic materials, such as copper or aluminum [9]. However, there are specific scenarios in which the use of metals may be undesirable. These situations include corrosive environments, applications in the field of biology where biocompatible materials are required, wearable electronics where flexibility and lightweight properties are critical, and electronic applications where electrical conductivity cannot guarantee safety conditions. Indeed, a heat sink with electrical insulation capability can effectively cool heat-generating components on an electronic board without the risk of a short circuit [10,11].

A potential solution to these challenges could involve substituting metals with polymeric materials [12]. Polymers have caught increasing attention in a wide range of industrial applications. In electronics, for instance, they find widespread use in various applications, including encapsulation, flip-chip technology, packaging, thermal interface materials, light-emitting diodes, solar cells, flexible electronics, and so on [13]. This spreading arises from their diverse functionalities, including tunable mechanical properties, high electrical resistivity and breakdown strength, adhesion, resistance to corrosion and fouling, excellent processability, cost-effectiveness, and lightweight nature [14,15,16]. However, polymers fail to meet the pressing demand for efficient heat dissipation due to their inherently low thermal conductivity, typically ranging from 0.1 to 0.5 W m^−1^ K^−1^, attributed to their amorphous structure. This limitation somewhat restricts their application in thermal management [17,18].

The need for materials combining the above-mentioned properties, such as thermal, mechanical, and electrical properties along with adequate processability and lightweight characteristics, has led to the study of composite materials having polymeric matrices. The fillers explored can be grouped into three categories: carbon-based materials [19,20], metals [21], and ceramics [22]. To maintain high electrical insulating properties, ceramic fillers must be used. Among the ceramic fillers, the most widely explored ones include: carbides, such as carbide-based MXenes [16] and silicon carbide (SiC) [23,24]; oxides, such as silicon oxide (SiO_2_) [25,26], aluminum oxide (Al_2_O_3_) [27], and magnesium oxide (MgO) [28,29]; and nitrides, such as aluminum nitride (AlN) [30,31] and boron nitride (BN) [32,33,34]. Among these, hexagonal boron nitride (BN) is considered highly promising [35,36,37]. BN possesses several favorable properties, such as low density (2.1 g cm^−3^), very high dielectric breakdown strength (~35 kV mm^−1^), and a low dielectric constant [38,39]. Due to its chemical structure, it exhibits anisotropic properties, with a higher thermal conductivity along the in-plane direction (~600 W m^−1^ K^−1^) compared to the out-of-plane direction (~30 W m^−1^ K^−1^).

Among the possible processing technologies for polymer matrix composites, Additive Manufacturing (AM) technologies are being investigated due to their advantages. AM allows for the construction of customized and topological structures with high flexibility, shortening the production cycle and reducing costs and materials waste [40,41]. In recent years, extrusion-based AM technologies, such as Fusion Deposition Modelling (FDM), have been reported as a facile method for processing polymer matrix composites to achieve highly oriented fillers. This orientation is achieved through shear forces within the nozzle during the printing process, which aligns the fillers along the printing direction. Therefore, this advantage of AM can be further used as a strategy to construct engineered materials for highly filled composites with designed performance [42,43]. Combining all these advantages, the study of heat management devices made of polymer matrix composites, such as heat sinks, has been rising in the last few years [44].

In this field, notable works include Kalsoom et al. [45], who, in 2016, developed a composite made of an acrylate-based polymer and synthetic microdiamond with an unknown thermal conductivity. The composite was processed through vat photopolymerization to print a heat sink model. A similar approach was used by Schleifer and Regev [46], who, in 2023, developed a composite made of acrylate-based polymer and graphene for vat photopolymerization. It reached a thermal conductivity of 0.39 W m^−1^ K^−1^, and its use as a heat sink was proposed. Nguyen et al. [47], in 2018, formulated a free-standing carbon nanotube buckypaper. It was then embedded in a heat sink made of a hybrid composite paste consisting of epoxy with graphite nanoplatelets and carbon fibers, processed by direct ink writing.

Focusing on FDM technology, in 2019, Waheed et al. [48] tailored a composite filament made of acrylonitrile-butadiene-styrene and synthetic microdiamond, achieving an in-plane thermal conductivity of 0.94 W m^−1^ K^−1^. With this material, they 3D-printed heat sinks, demonstrating enhanced thermal dissipation. In 2022, Huttunen et al. [49] focused on heat sink topology optimization exploiting FDM flexibility in producing complex geometries, using a commercial polyamide-based composite. However, to the best of our knowledge, the development of thermally conductive and electrically insulating polymer-based FDM 3D-printed heat sinks has been rarely investigated. It is possible to mention only Akintola et al. [50], who, in 2021, developed a composite filament for FDM made of polystyrene and BN nanotubes, achieving a thermal conductivity of 0.38 W m^−1^ K^−1^, and focusing on possible applications as heat sinks.

In our work, we characterized two thermoplastic polyamide (PA)-based composites, incorporating hexagonal BN and MgO as fillers, and investigated their morphological, thermal, electrical, and mechanical properties. This investigation demonstrated the achievement of a high in-plane thermal conductivity of 1.97 W m^−1^ K^−1^ and included an investigation of the effects caused by the chosen FDM processing technology. Furthermore, we explored the potential application of such BN-filled thermoplastic composites as lightweight heat sinks. This study demonstrates the feasibility of 3D printing heat sinks using BN microplatelets polymer composites with excellent thermal performance. An example of a heat management application, i.e., a pin fin heat sink, is presented and tested here as a proof of concept. For the first time, this study also explores thermal performances, using various heating sources, of electrically insulating heat sinks composed of BN-filled PA and produced by FDM.

## 2. Materials and Methods

### 2.1. Materials

Laticonther SP4 AM CP1/30 (PA–32BN) and Laticonther SP4 AM CP1/300 (PA–32BN–MgO) materials were developed and optimized by LATI S.p.A. (Vedano Olona, Italy). Both materials are ceramic-filled polymeric white composites with a polyamide (PA) matrix. The neat PA was characterized by Young’s modulus of 3.2 GPa, a breaking strength of 45 MPa, and elongation at break exceeding 100%. PA–32BN contains BN microplatelets only, whereas PA–32BN–MgO includes both BN microplatelets and MgO. Additionally, a commercially available black polylactic acid (PLA) filament with a diameter of 1.75 ± 0.05 mm was purchased from Maip Compounding S.r.l. (Torino, Italy). The latter has a thermal conductivity approximately ranging between 0.16 and 0.24 W m^−1^ K^−1^ [51,52,53], values that are comparable to PA’s thermal conductivity (which is between 0.12 and 0.28 W m^−1^ K^−1^ [54,55,56]), and served as a plain polymer reference. Its excellent printability allowed the printing of heat sinks for thermal performance comparison. 

### 2.2. Materials Processing through FDM 3D Printing

The materials in the form of filaments with a diameter of 1.75 ± 0.05 mm were dried in an oven at 90 °C for 24 h before being processed. The following printing parameters were selected after a preliminary screening: printing speed of 45 mm s^−1^; nozzle diameter of 600 µm; layer height of 300 µm; 100% infill; a closed chamber environment; bed temperature of 90 °C for PA–32BN and PA–32BN–MgO, and 60 °C for PLA; and nozzle temperature of 250 °C for PA–32BN and PA–32BN–MgO, and 210 °C for PLA. A Prusa i3 MK3S 3D printer (Prusa Research a.s., Prague, Czech Republic) was utilized to produce both the tested specimens for materials characterization and pin fin heat sinks via FDM. This involved generating a G-code file using the open-source slicing software PrusaSlicer 2.5.0 (Prusa Research a.s., Prague, Czech Republic).

### 2.3. Materials Characterization

X-ray diffraction (XRD) was performed using a Bruker D8 diffractometer (Bruker, Billerica, MA, USA) with a Cu Kα radiation source to characterize the crystalline structure, composition, and filler orientation in the printed composite materials. The solid samples fabricated by FDM were scanned at a speed of 1 s step^−1^, with steps of 0.02° and covering a 2*θ* range of 10–70°.

Scanning Electron Microscopy (SEM) was performed with an extended-pressure SEM Zeiss EVO 50 EP (Carl Zeiss S.p.A., Milano, Italy) to evaluate the printed composite materials’ microstructure. The specimens were obtained by FDM and then subjected to a brittle failure in a liquid nitrogen atmosphere.

Thermogravimetric analysis (TGA) was performed with a TA Instruments Q500 (TA Instruments, Inc., New Castle, DE, USA) under a nitrogen atmosphere. The samples (∼15–20 mg) were heated from 25 to 800 °C at a heating rate of 10 °C min^−1^ to evaluate both thermal stability and the filler content of the composite materials.

Non-isothermal differential scanning calorimetry (DSC) analyses were carried out using a Mettler-Toledo DSC/823e (Mettler Toledo, Columbus, OH, USA) instrument under a nitrogen atmosphere. Samples of around 15–20 mg underwent a heating ramp from 25 °C to 300 °C, followed by a cooling step from 300 °C to 25 °C, and subsequent heating from 25 °C to 300 °C, with a heating/cooling rate of 10 °C min^−1^. Cooling and the second subsequent heating ramps in this test were used to determine the characteristic temperatures and the degree of crystallinity, which was calculated according to the literature as follows:

(1) $$\chi =\frac{\Delta H_{polymer}}{\Delta H_{100\%}}\cdot 100$$

(2) $$\Delta H_{polymer}=\Delta H_{sample}\cdot \left(\frac{1}{1-\frac{w_{filler}}{100}}\right)$$

where $\Delta H_{sample}$ is the enthalpy of the composite melting peak, $\Delta H_{polymer}$ is the melting enthalpy of the polyamide in the sample, and $w_{filler}$ the weight percentage of filler content. The degree of crystallinity (χ) considers the melting enthalpy for the 100% crystalline polyamide, $\Delta H_{100\%}$= 230 J g^−1^ [57].

The thermal conductivity (k) was calculated for samples fabricated by FDM with the printing parameters reported in Section 2.2., using the following equation:

(3) $$k=\rho c_{p}\alpha$$

where *ρ* is the density of the sample, *c_p_* is the specific heat capacity, and *α* is the thermal diffusivity. The *ρ* was measured by Archimede’s method (ISO 1183-1) [58]. The *c_p_* of composites was measured with a Mettler-Toledo DSC/823e (Mettler Toledo, Columbus, OH, USA) instrument under a nitrogen atmosphere via a three-step method: (i) isothermal step at 15 °C for 3 min; (ii) linear ramp from 15 to 35 °C at 2 °C min^−1^; and (iii) isothermal step at 35 °C for 3 min. The α was measured at 25 °C via laser flash technique with LFA 467 Hyperflash (Netzsch, Selb, Germany) according to ASTM E-1461 standard [59]. The measurements were performed on samples with a squared section (12.7 mm × 12.7 mm) and a thickness of approximately 2 mm.

Electrical measurements were performed using a Keithley 6487 digital source-measure unit (Keithley Instruments, Inc., Solon, OH, USA) coupled with a probe measurement system applying 100 V. Both surface and volume resistivity were calculated from resistance measurements. Tests were carried out on samples fabricated by FDM with the printing parameters reported in Section 2.2. These samples had a circular section (diameter = 8 mm) and a thickness of approximately 3 mm.

Uniaxial tensile tests were performed following the ASTM D638-14 standard [60], utilizing a Zwick Roell Z010 dynamometer (ZwickRoell GmbH & Co. KG, Ulm, Germany) equipped with a 10 kN load cell. The tests employed a displacement rate of 10 mm min^−1^. Deformation values were tracked using a long-stroke extensometer. The mechanical anisotropy induced by three distinct raster orientations was verified by testing 3 mm thick dog-bone-shaped specimens produced via FDM for each material. The printing parameters for the dog-bone-shaped specimens are provided in Section 2.2. Additionally, one perimeter was included.

### 2.4. Preparation and Characterization of 3D-Printed Heat Sinks

A pin fin heat sink structure (diameter = 80 mm, height = 32 mm) featuring conical pins (top diameter = 3 mm, base diameter = 9 mm) was designed using computer-aided design (CAD). This structure was chosen as a proof of concept, as pin fin heat sinks are a common thermal management solution employed to dissipate heat from electronic components. Commonly employed for anisotropic materials, the pins’ structure facilitates uniform heat dissipation, driven by effective convection-based heat transfer. As heat travels through the pins, it efficiently cools the electronic device.

The thermal efficiency of the printed structures was assessed by subjecting the pin fin heat sinks to two distinct heating procedures. Two separate heat sources were employed: (i) a heating plate IKA C–MAG HS 7 (IKA-Werke GmbH & Co., Staufen, Germany) set to 90 °C, onto which the pin fin heat sink was placed for conduction-based heating; and (ii) an infrared (IR) lamp Philips IR250 (Philips SpA, Eindhoven, The Netherlands) with a power of 250 W, positioned 0.3 m away from the bottom of the pin fin heat sink, used for irradiation-based heating. Thermal performance was evaluated by capturing IR images at 20-s intervals over 20 min during the heating processes. Microbolometric IR cameras, specifically the FLIR T1020 and FLIR B6 models (Teledyne FLIR LLC, Wilsonville, OR, USA), were employed to capture the frontal and top views, respectively. Data processing was conducted using Grayess^®^ IRT Analyzer 7 (GRAYESS, Inc., Bradenton, FL, USA).

## 3. Results and Discussion

### 3.1. Materials Characterization

This section presents the characterization of the BN microplatelets composite materials Laticonther SP4 AM CP1/30 (PA–32BN) and Laticonther SP4 AM CP1/300 (PA–32BN–MgO). It will cover morphological and compositional analyses of the filaments, as well as a discussion of the thermal, electrical, and mechanical properties of the printed materials.

#### 3.1.1. Morphological Analyses

**Figure 1:**
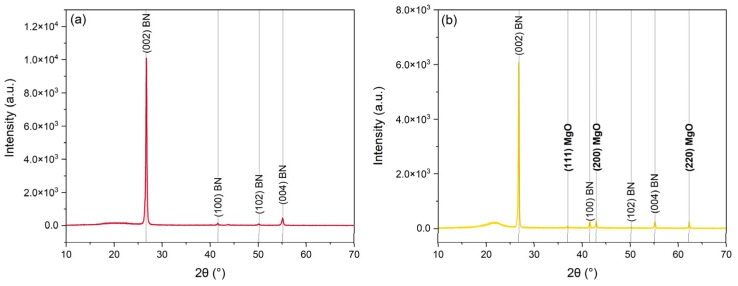
Diffractograms of 3D-printed PA–32BN (**a**) and PA–32BN–MgO (**b**) highlighting the peaks associated with the crystalline planes of BN and MgO.

In Figure 1, the XRD diffractograms of the 3D-printed composites are presented to qualitatively confirm the alignment of BN platelets.

The diffraction peaks for BN, namely (002) and (100), correspond to the horizontal direction (i.e., the printing direction) and vertical direction (i.e., the direction perpendicular to the printing direction), respectively. For this reason, the ratio between the intensity of these diffraction peaks can be an indication of the particle orientation. Their evaluation also allows a quantitative estimation of an orientation degree (OD), which according to the existing literature [61] can be defined as:

(4) $$OD=\frac{I_{002}}{I_{002}+I_{100}}$$

where $I_{002}$ and $I_{100}$ are the intensities of the (002) and (100) diffraction peaks in the diffractograms, respectively. After fitting the peaks using Gaussian curves, $I_{002}$ and $I_{100}$ were calculated for both PA–32BN and PA–32BN–MgO. The $I_{002}$/$I_{100}$ ratios were equal to 70 and 25 for PA–32BN and PA–32BN–MgO, respectively. The calculated OD values were 99% for PA–32BN and 96% for PA–32BN–MgO. This implies that in PA–32BN, BN platelets exhibit better alignment along the printing direction compared to PA–32BN–MgO. This outcome indicates that the presence of MgO particles impeded the BN alignment during the various alignment processes, including filament fabrication via extrusion and component processing through FDM. Since FDM is a 3D printing technology based on extrusion principles, this suggests that MgO particles interfered with the alignment. Consequently, PA–32BN is expected to display more evident anisotropy in its properties. On the other hand, the inclusion of MgO particles in the compounding could potentially yield more uniform properties across different directions.

**Figure 2:**
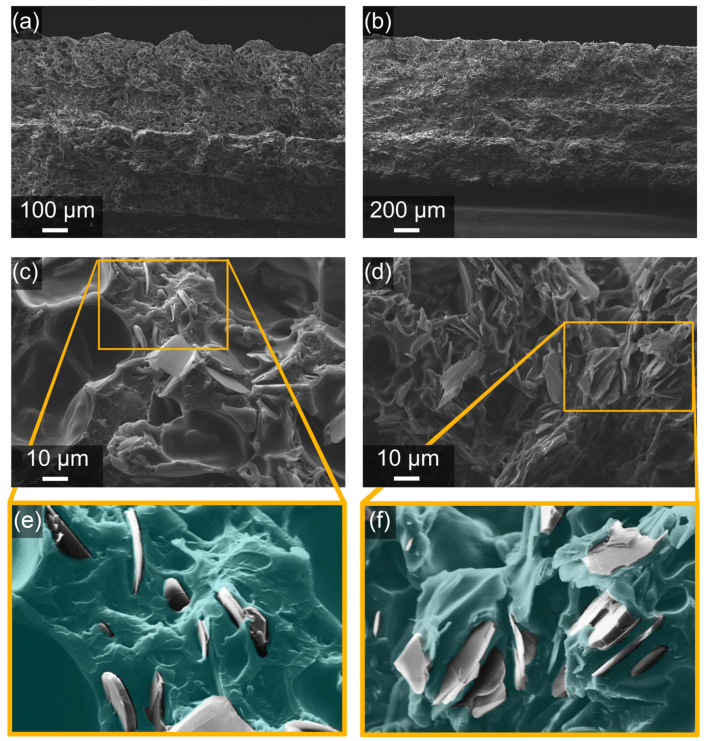
Cross-section images by Scanning Electron Microscopy of 3D-printed PA–32BN at magnification 150× (**a**) and 2500× (**c**); PA–32BN–MgO at magnification 75× (**b**) and 1500× (**d**). Modified images with colors to highlight fillers in PA–32BN (**e**) and PA–32BN–MgO (**f**).

In Figure 2, SEM cross-section images of 3D-printed PA–32BN and PA–32BN–MgO are shown. Both materials exhibit consistent patterns, showing even fractured surfaces devoid of defects at the millimeter scale, along with uninterrupted continuity among successive fused filament depositions. At higher magnification, micrometric BN platelets are visible, displaying their disc-like morphology with a thickness < 1 µm and an average lateral dimension on the order of 10 µm. A certain orientation of BN platelets can be observed, as highlighted in Figure 2e,f, where the particles tend to show their thickness and align along the printing direction. Due to their relatively low volume content in the composites and effective dispersion, they are likely to exhibit low packing density, resulting in poor filler–filler contacts for both PA–32BN and PA–32BN–MgO.

#### 3.1.2. Thermal Properties

**Figure 3:**
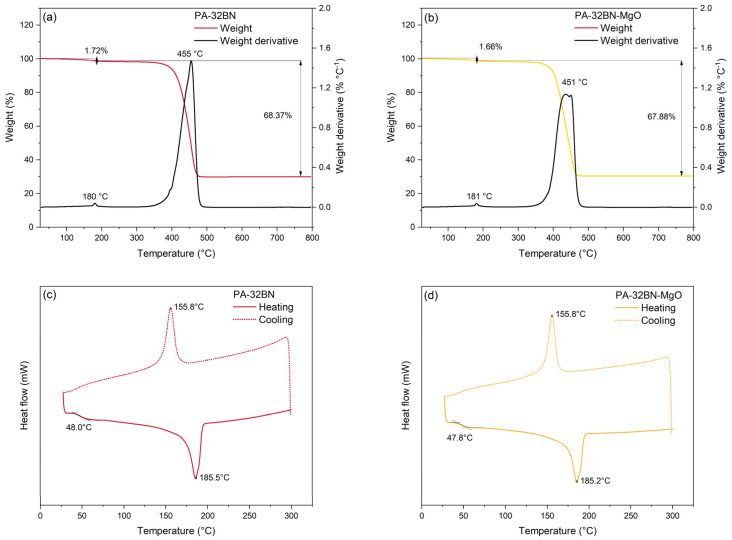
Thermal properties of PA–32BN and PA–32BN–MgO analyzed through TGA (**a**,**b**) and DSC (**c**,**d**).

Figure 3a,b display the TGA analysis for PA–32BN and PA–32BN–MgO, respectively. The materials underwent a comparable thermal degradation process. Following an initial weight loss of approximately 1.7 wt% around 180 °C, attributed to humidity desorption and degradation of volatile monomers and oligomers, the thermal degradation of the polymeric matrices commenced at approximately 350 °C [62]. The peak weight loss occurred at roughly 450 °C. These values align with the typical behavior of polyamides and are minimally impacted by the presence of ceramic fillers [56,63]. Beyond 500 °C, the sample weight is associated with the residual ceramic filler and remained constant up to 800 °C. The filler content was estimated to be approximately 32 wt% for both PA–32BN and PA–32BN–MgO.

Figure 3c,d exhibit the DSC analysis for PA–32BN and PA–32BN–MgO, respectively. The materials showed analogous characteristic temperatures to those of the polyamide matrices. The glass transition temperature (*T_g_*) was 48 °C; the melting temperature (*T_m_*) was 185 °C, with an associated degree of crystallinity (*χ*) of 28%; and the crystallization temperature during the cooling (*T_c_*) was 156 °C.

#### 3.1.3. Mechanical Properties

Since mechanical properties can be significantly influenced by printing and structural parameters [64], three distinct raster orientations were investigated in in-plane printed dog-bone-shaped specimens. This exploration is essential due to the anisotropic mechanical properties of components produced through FDM, which depend on the printing direction.

**Figure 4:**
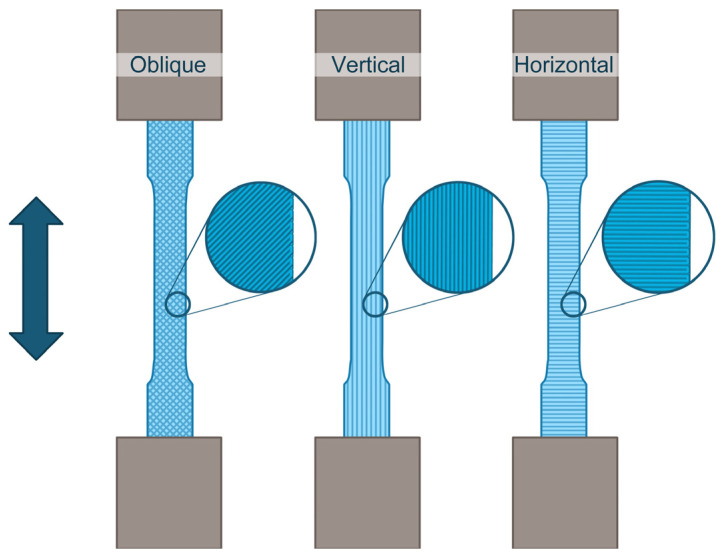
Schematic illustration of the dog-bone-shaped specimens for tensile tests, fabricated by FDM with three different raster orientations to the applied force: oblique (±45°), vertical, and horizontal (from left to right).

As illustrated in Figure 4, the dog-bone-shaped specimens were fabricated examining the effect of depositing the fused filaments with three distinct raster orientations concerning the direction of the applied force during the tensile tests: (i) oblique orientation, signifying filaments were positioned at ±45° to the applied force direction during tensile tests; (ii) vertical orientation, meaning filaments were laid parallel to the applied force; and (iii) horizontal orientation, indicating filaments were placed perpendicular to the applied force.

Table 1 presents Young’s modulus, ultimate tensile strength (UTS), and strain at break derived from the stress-strain curves for both PA–32BN and PA–32BN–MgO. PA–32BN exhibited Young’s modulus, UTS, and strain at break within the ranges of 2626–3158 MPa, 21.4–30.4 MPa, and 3.4–4.5%, respectively. Correspondingly, PA–32BN–MgO demonstrated similar values, with Young’s modulus, UTS, and strain at break falling within the ranges of 2569–2944 MPa, 30.7–34.3 MPa, and 7.3–8.2%, respectively. Both materials exhibited brittle failure, as anticipated due to their high filler content.

Regarding the raster orientation, mechanical properties decreased progressively from the vertical direction to the oblique (±45°) and horizontal directions. This trend is consistent with the prior literature [65]. This phenomenon can be explained by considering the interlayer junctions, which can reduce the strength of the sample due to the increased influence of the bonding effect, at the expense of the material properties. These junctions, when oriented perpendicularly to the force, lead to lower load-bearing capacities and reduced mechanical properties. On the other hand, if the junctions are oriented parallel to the applied force during tensile tests, their effects on the mechanical properties are relatively minor.

However, this study revealed that PA–32BN and PA–32BN–MgO displayed mild anisotropy in their mechanical properties across the tested directions. This outcome implies excellent interaction among the deposited filaments, which was facilitated by the closed-chamber system allowing gradual cooling of the material. Furthermore, it is essential to note the favorable filament packing morphology, which exhibits minimal void and defects at their interlayer junctions, as confirmed by the SEM images of the 3D-printed samples section (Figure 2a,b). Additionally, it must be considered that, regardless of the raster orientation, one perimeter was included in the dog-bone-shaped specimens. This factor can significantly influence the overall mechanical properties, especially in the horizontal and oblique cases. In conclusion, it is worth reminding that in all three cases, despite the varying orientation of the deposited fused filaments, BN platelets were always aligned within the printing plane, as previously discussed in the morphological analysis. Therefore, BN platelets had an impact on the mechanical properties, but with small effects on the raster orientation tested.

#### 3.1.4. Thermal and Electrical Conductivities

The thermal conductivities of PA–32BN and PA–32BN–MgO were assessed both in-plane (*k_//_*), i.e., along the direction of the filler orientation, and through-plane (*k_⊥_*), i.e., perpendicular to that direction, using laser flash analysis (LFA), according to a well-established method [66]. Electrical resistivities were measured for both surface (*ρ_s_*) and volume (*ρ_v_*). The calculated thermal conductivities and the measured electrical resistivities are presented in Table 2.

In the direction of filler orientation, i.e., the printing direction, PA–32BN and PA–32BN–MgO exhibited thermal conductivities of 1.97 ± 0.11 W m^−1^ K^−1^ and 1.22 ± 0.05 W m^−1^ K^−1^, respectively. Conversely, in the orthogonal direction, PA–32BN and PA–32BN–MgO displayed thermal conductivities of 0.69 ± 0.01 W m^−1^ K^−1^ and 0.64 ± 0.02 W m^−1^ K^−1^.

The fillers are effective in significantly increasing the thermal conductivities by up to an order of magnitude compared to the polymer matrix, which is documented in the literature to fall within the range of 0.12–0.28 W m^−1^ K^−1^ [54,55,56]. Owing to the anisotropic morphology of BN, characterized by platelets as shown in Figure 2, the composites exhibit pronounced thermal conductivity anisotropy. Furthermore, this anisotropy is established during the printing process in extrusion-based technologies like FDM, owing to the shear stresses generated within the nozzle [42,43,67]. The high values achieved can be attributed to the high in-plane direction thermal conductivity of BN platelets, which is about 600 W m^−1^ K^−1^, due to the strong covalent bonds between B atoms and N atoms and the effective establishment of a thermal pathway formed by BN microplatelets oriented along the printing direction [68,69,70]. However, the ratio *k_//_*/*k_⊥_* for PA–32BN was 2.8, significantly higher than that of PA–32BN–MgO, which was 1.9. This behavior confirms that the introduction of MgO particles hindered the alignment of BN particles, as evaluated by XRD, and could diminish the overall anisotropy of the properties.

Both PA–32BN and PA–32BN–MgO displayed volume electrical resistivity in the order of magnitude of 10^12^ Ω cm, confirming the highly electrically insulating behavior of the composites.

The thermal conductive and electrically insulating properties of PA–32BN and PA–32BN–MgO are higher than those mainly reported in the literature and are coherent with the most recent results reported for 3D printable materials by FDM. In Table 3, a comparison among thermally conductive and electrically insulating filaments, including BN as filler, is presented. It highlights the polymeric matrix, filler content, and the achieved values of thermal conductivity. As summarized, there are only a few polymeric filaments for FDM technology loaded with BN platelets, and only some of them have *k_//_* between 1 and 2 W m^−1^ K^−1^. Specifically referring to filaments with a PA matrix, PA–32BN seems to be the best compromise. It reached an in-plane thermal conductivity comparable to the work presented by Geng et al. [67], but at a lower weight filler content, thus with less impact on the mechanical properties.

### 3.2. Thermal Performances of 3D-Printed Heat Sinks

The clear trend of electronic devices becoming smaller and more powerful has increased the demand for efficient heat dissipation, which is crucial for optimal device operation. Heat sinks play a crucial role by effectively dissipating excess heat, thereby enhancing overall device performance and functionality. Furthermore, AM technologies offer the potential to design complex structures for customized thermal devices with optimized shapes.

To address these considerations, a pin fin heat sink was conceptualized and produced using FDM with PA–32BN, PA–32BN–MgO, and a plain polymer with a reference thermal conductivity of 0.13 W m^−1^ K^−1^ [75]. Subsequently, IR thermography was employed to qualitatively assess the heat sinks’ thermal performance and compare them with the thermal conduction properties of the materials.

#### 3.2.1. Modeling and Fabrication by FDM

**Figure 5:**
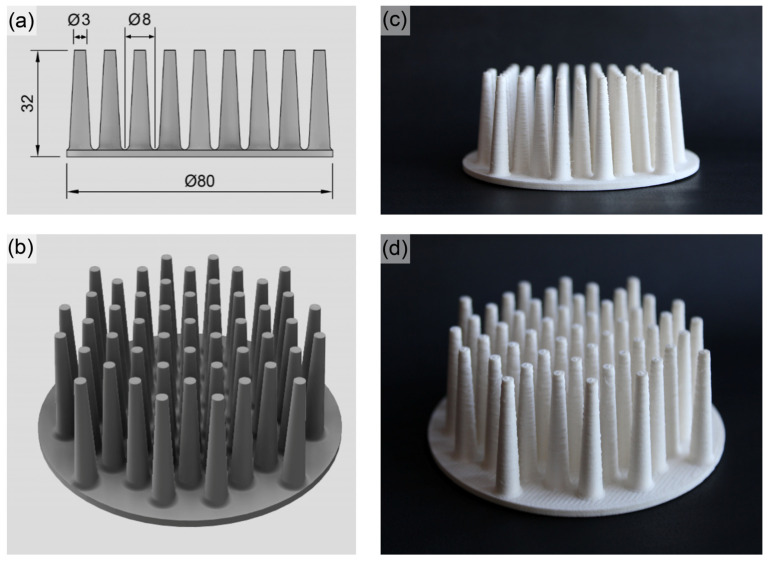
Technical drawing of the pin fin heat sink (**a**), 3D CAD model (**b**), and the 3D-printed FDM-manufactured version (**c**,**d**).

As shown in Figure 5, a centrosymmetric pin fin heat sink was designed and fabricated by FDM. Despite the high content of ceramic microfillers, a successful printing of the heat sinks without nozzle clogging was achieved. This resulted in high-quality components with accurate dimensions, as shown in Figure 5c,d.

#### 3.2.2. IR Thermography

The thermal performance of the printed pin fin heat sinks was assessed using two distinct heating methods. In the former approach, a heating plate was utilized, and the pin fin heat sink was placed in contact with it to facilitate heating through conduction. In the latter experiment method, an IR lamp positioned under the pin fin heat sink was employed to induce heating through irradiation.

##### Conductive Heating Source

**Figure 6:**
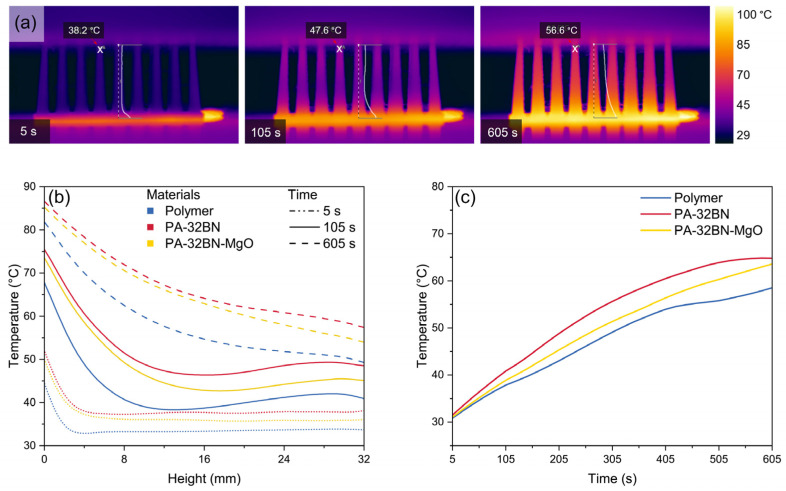
(**a**) Thermograms of PA–32BN pin fin heat sink heated by a conductive system after 5 s, 105 s, and 605 s. (**b**) Temperature profiles as a function of the pin height of pin fin heat sinks heated by a conductive system, comparing plain polymer, PA–32BN, and PA–32BN–MgO at various times. (**c**) Temperature profiles as a function of time at 16mm height on pin fin heat sinks heated by a conductive system, comparing plain polymer, PA–32BN, and PA–32BN–MgO.

To assess thermal performance using a conductive heat source, the pin fin heat sink was placed in contact with a hot plate for 10 min (Supporting Information, Video S1). The resultant conduction-based heating was monitored using a thermal camera, which provided temperature readings for the points on the surface, as shown in Figure 6a.

For a qualitative analysis, temperature line profiles were plotted. Figure 6b illustrates the temperature line profiles as a function of the height of the pin, from the contact surface (0 mm) to the top of the pin (distance 32 mm) at 5, 105, and 605 s. These profiles belong to the three materials: the plain polymer; PA–32BN; and PA–32BN–MgO.

Among all the materials under investigation, the temperature along the pin naturally rose over time, due to the heat source positioned at the base of the heat sink. The temperature was at its maximum near the vicinity of the heat source and gradually decreased by increasing the distance from the heat source. By comparing the materials’ performances after 10 min, both PA–32BN and PA–32BN–MgO exhibited similar temperature profiles. Moreover, temperature values measured for BN-filled and 3D-printed polymers were always higher than the temperatures recorded for the plain polymer, which has the lowest temperature. Specifically, at a distance of 32 mm from the heating plate after 10 min, temperature values measured for the plain polymer, PA–32BN–MgO, and PA–32BN were 49.5 °C, 54.2 °C, and 57.6 °C, respectively. This trend indicated the superior efficiency of PA–32BN in effectively transferring heat compared to the other materials. Notably, PA–32BN reached the temperature set by the heating source more rapidly, featuring a substantial temperature discrepancy of 8.1 °C compared to the plain polymer after 10 min. These results were confirmed by the temperature profiles as a function of time shown in Figure 6c, which plots the temperature at the midpoint (16 mm) of the heat sinks for the three materials.

These findings are in agreement with the thermal conductivity properties of the materials. Specifically, PA–32BN exhibits a higher thermal conductivity of 1.97 W m^−1^ K^−1^ in-plane, and 0.69 W m^−1^ K^−1^ out-of-plane. This surpasses both the intermediate thermal conductivity of PA–32BN–MgO, which has 1.22 W m^−1^ K^−1^ in-plane, and 0.64 W m^−1^ K^−1^ out-of-plane, and the known isotropic thermal conductivity of 0.13 W m^−1^ K^−1^ of the plain polymer.

##### Irradiative Heating Source

**Figure 7:**
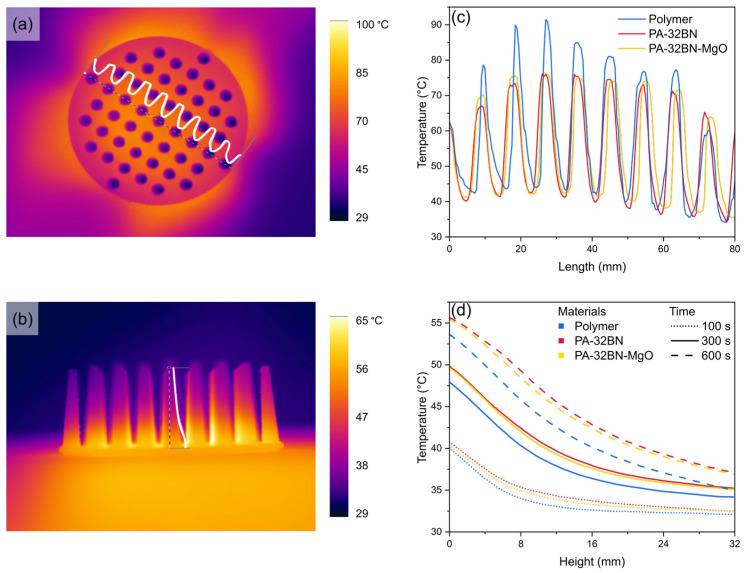
Thermograms from both top (**a**) and frontal (**b**) viewpoints of PA–32BN pin fin heat sink heated by an irradiation system, showing highlighted temperature profile curves. (**c**) Temperature profiles along the diameter of the heat sinks made of plain polymer, PA–32BN, and PA–32BN–MgO at 600 s, extrapolated from the top viewpoint. (**d**) Temperature profiles along the pin height of pin fin heat sinks heated by an irradiation system, comparing plain polymer, PA–32BN, and PA–32BN–MgO at various times, extrapolated from the frontal viewpoint.

For a comprehensive evaluation of thermal performance, the effect of an IR lamp positioned under the base of the pin fin heat sink as an irradiative heating source was examined for 10 min (Supporting Information, Video S2). In this context, two thermal cameras were employed to capture IR images from both the top (Figure 7a) and the front view (Figure 7b).

The images acquired from the top view facilitated an assessment of the heating process occurring at the lower section of the heat sink. This phenomenon was primarily attributed to irradiation, owing to the direct exposure of the heat sink’s base to the IR lamp. Conversely, the images obtained from the frontal view were utilized to analyze the heating process along the heat sink’s pins. This mode of heating was predominantly driven by conduction, as a result of the transfer of heat within the material from the base to the upper region.

Figure 7a illustrates the thermograms of the heat sink observed from a top view. It highlights the occurrence of elevated temperatures at the base of the heat sink, whereas the upper ends of the pins exhibited notably lower temperatures. To evaluate the temperature achieved at the heat sink’s base, temperature profiles are presented in Figure 7c after 600 s of heating for the three materials under investigation. In the curves, temperature peaks correspond to the heat sink base, while the valleys are attributed to the upper ends of the pins, where the temperature is the minimum since the heating source was positioned under the heat sink.

Comparing the curves of the three materials, it is evident that the composites exhibited a similar behavior. In fact, the maximum temperatures achieved at the base of the heat sink are 75.8 and 75.2 °C for PA–32BN and PA–32BN–MgO, respectively. Conversely, the plain black polymer showed higher inhomogeneities in the temperature at the base, with temperatures reaching 91.3 °C. This behavior finds its explanation in the influence of color on irradiation phenomena. Indeed, the composite materials PA–32BN and PA–32BN–MgO exhibited a white appearance, while the plain polymer was black, a color that absorbs heat more readily through irradiation, due to its higher absorptivity [76]. The choice of the black plain polymer enabled a comparison of thermal performances under more challenging conditions, allowing us to study how the larger quantity of absorbed heat was transferred by conduction within the pins of the plain polymer heat sinks, compared to those made of composites.

The thermograms acquired from the frontal view (Figure 7b) were analyzed, and temperature line profiles along the height of the pin, from the contact surface (0 mm) to the top of the pin (distance 32 mm) at times of 5, 105, and 605 s were plotted for the plain polymer, PA–32BN, and PA–32BN–MgO. The general trend corresponds to that observed in the conduction experiment. By comparing the performances of the materials after 600 s, both PA–32BN and PA–32BN–MgO exhibited similar temperatures and they exceeded the temperature recorded for the plain polymer, which exhibited a lower temperature. Specifically, at the top of the pin (distance of 32 mm), the temperatures measured at a time of 600 s are 35.2 °C for the plain polymer, 37.3 °C for PA–32BN–MgO, and 37.4 °C for PA–32BN (Figure 7d).

These results highlight the inefficiency of the plain polymer in heat dissipation, given its thermal conductivity, which is one order of magnitude lower than that of the composites. Despite the plain polymer’s ability to absorb more heat due to its black color (i.e., higher absorptivity), the heating experiment revealed an inadequate distribution of heat along the pins of the heat sink. Instead, heat accumulated predominantly at the base due to the polymer’s low thermal conductivity. In contrast, the composites efficiently distributed the heat along the height of the pins, resulting in higher temperatures at the upper part compared to the plain polymer.

This observed trend, consistent with the conduction experiment and the thermal properties of the materials, highlights the remarkable heat transfer efficiency by conduction of the composite materials when compared to the plain polymer. Additionally, it is important to consider that for practical applications of heat management, the inherent white color of polymer composites containing BN microparticles could be modified by dispersing a minimal amount of carbon black particles. This incorporation would raise the composite emissivity from 0.7 to 0.9, thereby further enhancing the thermal performance of devices.

In conclusion, thermographic tests confirm the advantageous impact of BN microplatelet incorporation in enhancing thermal conduction and, consequently, the thermal performance of devices. This renders the polymer-based materials suitable for lightweight heat sink applications.

## 4. Conclusions

In this study, innovative BN microplatelet composites based on 3D printable polyamide-based thermoplastics polymers, specifically designed to combine both thermal conduction and electrical insulation properties for FDM technology, were thoroughly examined. The investigation encompassed morphological, mechanical, thermal, and electrical properties, revealing structure–property relationships, for instance between a preferential orientation of BN microplatelets and composite thermal conductivity as well as between their morphological structure and mechanical properties.

Notably, the influence of different microfillers, with a specific focus on BN platelets and their combination with MgO particles, was explored. The observed structural effects on 3D-printed material, where the presence of MgO could impact BN alignment within the polymeric matrix during processing, suggested a complex interplay between components, resulting in significantly different thermal properties among the composites. PA–32BN, incorporating 32 wt% of BN microplatelets, exhibited higher filler orientation along the printing direction, as indicated by morphological analysis, leading to a remarkable in-plane thermal conductivity of 1.97 W m^−1^ K^−1^, with a ratio between the in-plane and out-of-plane thermal conductivity *k_//_*/*k_⊥_* of 2.8. In contrast, PA–32BN–MgO, incorporating MgO particles, exhibited a lower filler orientation, resulting in more homogeneous thermal properties, with an in-plane thermal conductivity of 1.22 W m^−1^ K^−1^ and a ratio between the in-plane and out-of-plane thermal conductivity *k_//_*/*k_⊥_* of 1.9. Both composites displayed thermal conductivity values one order of magnitude higher compared to the polymers used. Additionally, excellent electrical insulative properties were found, with a volume electrical resistivity of the order of 10^12^ Ω cm, ensuring safety conditions for electronic applications. 

Furthermore, the potential applications of these highly thermally conductive, electrically insulating, and printable compositions were demonstrated. Specifically, lightweight pin fin heat sinks were conceptualized and developed using FDM 3D printing technology. A comprehensive assessment of their thermal performance was carried out through thermography, involving the analysis of responses under different heating conditions, and demonstrating interesting improvements for the investigated composites compared to polymeric materials.

The findings of this research unveil promising routes for the rapid and cost-effective fabrication of thermal devices, including heat sinks, utilizing extrusion-based AM technologies like FDM.

## Figures and Tables

**Table 1:** Mechanical properties assessed via tensile tests on 3D-printed dog-bone specimens of PA–32BN and PA–32BN–MgO with three different building orientations in the printing plane.

	Orientation	PA–32BN	PA–32BN–MgO
Young’s modulus [MPa]	Oblique	2865 ± 209	2861 ± 200
	Vertical	3158 ± 128	2944 ± 133
	Horizontal	2626 ± 277	2569 ± 164
Ultimate tensile strength [MPa]	Oblique	24.3 ± 0.5	34.3 ± 0.4
	Vertical	30.4 ± 1.5	34.1 ± 0.1
	Horizontal	21.4 ± 1.2	30.7 ± 0.4
Strain at break [MPa]	Oblique	3.5 ± 0.2	8.1 ± 0.9
	Vertical	4.5 ± 0.5	8.2 ± 0.9
	Horizontal	3.4 ± 0.3	7.3 ± 0.6

**Table 2:** In-plane (*k_//_*) and out-of-plane (*k_⊥_*) thermal conductivities, surface (*ρ_s_*), and volume (*ρ_v_*) electrical resistivities of PA–32BN and PA–32BN–MgO.

		PA–32BN	PA–32BN–MgO
Thermal conductivity	*k_//_* [W m^−1^ K^−1^]	1.97 ± 0.11	1.22 ± 0.05
	*k_⊥_* [W m^−1^ K^−1^]	0.69 ± 0.01	0.64 ± 0.02
Electrical resistivity	*ρ_s_* [Ω]	3.0 × 10^12^	5.0 × 10^12^
	*ρ_v_* [Ω cm]	1.7 × 10^12^	2.4 × 10^12^

**Table 3:** Comparison of composition and thermal conductivities of electrically insulating materials processed by FDM, as reported in the literature.

Matrix	BN Filler Content	Thermal Conductivity		Ref.
		*k_//_* [W m^−1^ K^−1^]	*k_⊥_* [W m^−1^ K^−1^]	
PA	32 wt%	1.97	0.69	This work
PA	(+MgO) 32 wt%	1.22	0.64	This work
ABS	15 wt%	0.37	-	[71]
TPU	30 wt%	1.51	1.26	[8]
ABS	35 wt%	0.93	~0.28	[72]
PLA	40 wt%	0.70	-	[73]
LLDPE	40 wt%	0.70	-	[73]
TPU	40 wt%	2.56	1.20	[42]
PA	45 wt%	1.52	0.96	[74]
PA	30 vol%	2.03	-	[67]

## Data Availability

The data that support the findings of this study are available from the corresponding author upon reasonable request.

## Supplementary Materials

The following supporting information can be downloaded at: https://www.mdpi.com/article/10.3390/polym16030432/s1, Video S1, Video S2.
